# New Developments at "The London": How the Growth of Work Is Being Met

**Published:** 1914-06-06

**Authors:** 


### NEW DEVELOPMENTS AT "THE LONDON."

### How the Growth of Work is being Met.

### The Anæsthetic Staff.

The quarterly report of the house committee drew attention to the fact that on account of the increasing number of surgical operations, which last year beat all records—now anæsthetics are given at the rate of about sixty every day—the committee have decided to appoint two extra clinical assistants as anæsthetists. The anæsthetic staff will then consist of five visiting anæsthetists and seven receiving-room officers, who are also called resident anæsthetists, and have to take their turn in giving anæsthetics.

Your committee have always considered that the administration of anæsthetics should be given only by highly skilled men, and, in fact, the committee drew attention to this in their last report. They are very gratified and proud to report that last year out of over 22,000 anæsthetics there were only seven cases of death, and most of these seven cases were patients who were *in extremis*, and for whom operation under an anæsthetic was a last resource.

### The Tuberculosis Arrangements.

With regard to tuberculosis no kind of definite arrangements have yet been made, for the question becomes more difficult the more it is studied. If, states the report, the object of the Act is to stamp tuberculosis out of the country, it is obvious that it was useless to attempt to do so (tuberculosis being an infectious disease) if only insured persons were treated; therefore the Government decided that uninsured, as well as insured, persons are to be treated, and this at once made the task a most difficult one. Arrangements have to be made for domiciliary treatment, for hospital treatment when the patient is acutely ill, for sanatorium treatment for such cases as are only slightly attacked and may hope for recovery, and with other institutions for advanced cases, really homes for the dying. The London Hospital, with the best intention of giving all the assistance it can, finds its work the more difficult because of the multitude of authorities who must be applied to—to the County Council for uninsured cases that need institutional treatment, to the London Insurance Committee for insured cases that need institutional treatment, to the Borough Councils with regard to cases, both insured and uninsured, who require home treatment, and now another authority is coming in, viz., the Metropolitan Asylums Board which, the committee believe, is to treat certain cases needing sanatorium treatment.

### The New Hostel.

The committee has signed the agreement with the City for the lease of a site close to the hospital, and which can be seen from its windows, where it is proposed to build a hostel for the resident doctors. This building is proposed to be connected with the hospital by a subway. In the meantime the committee have decided to erect a tin building in the grounds for the accommodation of the resident doctors during the building of this hostel, as the rooms now occupied by them are required for other purposes.

### Two New Aural Wards.

The committee has approved plans for the building of two new wards, in the wing where the quarterly court meets, for the treatment of aural cases. Considering the very large aural out-patient department (9,800 last year), the twenty beds which the London Hospital now has, and which are dotted about in different parts of the hospital, are felt to be totally inadequate for the needs of the East-End. Many serious cases of mastoid disease, which, undoubtedly, were there accommodation, ought to be admitted, have had to be sent away. The public appear to think that ear diseases are inconvenient, but not very important; it is now known, states the report, that many deaths occur through inflammation of the brain directly as the result of certain ear diseases. The new department, if ever it is built, will contain thirty-four beds. It is not generally known, and cannot be too widely known, that for the whole of East London there are no ear and throat beds. The authorities have given up these twenty beds from the surgeons. But this necessity for some aural beds is a very real one, and the committee are hoping that someone will help to build this much-needed ward.

### Laundry Enlargement.

The enlargement of the laundry is at present in hand. The laundry work has so increased that long since it had outgrown the capacity of the old laundry, and further machinery and more accommodation has to be provided. About 10,000 "pieces" a day pass through the laundry. The great care taken in modern operative surgery is largely responsible for this great increase; for instance, a single heavy afternoon's operating will demand 250 operating coats and 350 towels.

### The Wards for Syphilis.

With regard to the wards to be opened for the treatment of syphilis, the plans have been submitted to the Grocers' Company, who are the generous donors of these wards, and the actual work of building will very shortly commence, though the present dispute in the building trade may be a source of trouble.

### The Heart Department.

Certain alterations and extensions are also required in the heart department, a department which is doing a great amount of good under Dr. James Mackenzie and his assistant, Dr. John Parkinson. This department, of necessity, has to be in the basement; if on any other floor the vibration would affect the extremely delicate instruments used in recording heart beats. By certain of these instruments certain forms of heart disease have been diagnosed long before they could have been heard by the stethoscope.

X-ray Department.

The committee have sanctioned the purchase of apparatus which will cost £300 or £400 for giving what are known as massive filtered doses of *x*-ray. The report says some remarkable results have been obtained by these special rays in cases of cancer which were quite incurable. Photographs of cases before and after cure were brought before the committee, and as a result the committee ordered this apparatus.

[Note.—*We hope to publish next week the gist of Mr. W. T. Paulin's speech at this meeting.*]

